# Synthesis and characterization of novel thermoresponsive suspensions via physical adsorption of poly[di(ethylene glycol) methyl methacrylate] onto polystyrene microparticles

**DOI:** 10.1080/15685551.2023.2211356

**Published:** 2023-05-10

**Authors:** Azad Sadraddin

**Affiliations:** Chemistry Department, Education College, Salahaddin University, Iraqi kurdistan, Iraq

**Keywords:** Thermoresponsive, biomedical application, suspension, polystyrene particles, poly[di(ethylene glycol) methyl methacrylate]

## Abstract

Thermoreversible colloidal suspensions/gels have attracted recent research attention in the field of biomedical applications. In this study, a novel thermoresponsive particle suspension with thermoreversible gelation properties has been prepared for biomedical application. First, polystyrene (PS) microspheres were synthesized by dispersion polymerization and poly diethyleneglycolmethylmethacrylate (PDEGMA) polymer were synthesized via free radical polymerisation. Then, the new developed thermoresponsive suspensions were prepared via physical adsorption of a thermoresponsive polymer, poly[di (ethylene glycol) methyl methacrylate] (PDEGMA), onto the surface of polystyrene microspheres. PDEGMA acts as a steric stabilizer and induces thermoreversible gelation via chain extending and collapsing below and above its lower critical solution temperature (LCST), respectively. Scanning electron microscopy (SEM), ^1^H NMR spectroscopy, Gel permeation chromatography (GPC), UV-vis spectroscopy, Rheometric measurement were conducted to characterize the prepared particles, polymers and suspensions. SEM images show that monodisperse microspheres with the sizes range 1.5–3.5 μm were prepared. UV-vis measurements demonstrate thermoresponsive properties of PDEGMA. ^1^H NMR and GPC analysis confirms structural properties of prepared PDEGMA. Tube inversion tests demonstrated that the aqueous suspensions of the particles and polymer exhibited thermoreversible fluid-to-gel transitions. Rheological characterization revealed that the viscoelastic properties of the prepared suspension/gels can be fine tuned. This enables applications of the prepared gels as scaffolds for three-dimensional (3D) cell cultures.

## 1. Introduction

Due to their use in biomedical applications such as drug delivery, tissue regeneration, and organ engineering, physical adsorption is simple and rapid and can be adapted to many microparticles. Core-shell microparticles, which are gaseous bubbles, liquid or solid enclosed by a protective shell, are versatile carriers and have potential biomedical applications. The preparation and properties such as morphology, composition, configuration and size of core-shell particles have been extensively investigated [1–6], researchers have become interested in thermoreversible colloidal suspensions/gels. Long-range repulsions and the resulting particle separation result in the formation of colloidal suspensions that are stabilized [7]. Moreover, the microspheres were chosen because polystyrene surfaces (PS) have been widely used for in vitro cell culture applications and are approved by the Food and Drug Administration (FDA) for specific biomedical applications, as described previously.

The PDEGMA thermoresponsive component was chosen due to its low critical solution temperature (LCST, 26°C), which is slightly higher than room temperature but lower than human body temperature [8–10]. In addition, PDEGMA is resistant to protein absorption [11], which would support weak, reversible cell adhesion [12]. This protein resistance is crucial for a variety of surface engineering applications, including drug delivery and biomaterials [13]. According to Qin and Zaman [14], stable colloidal systems typically have particle mean sizes that range from a few nanometers to several micrometers, which distinguished contributions from hard-sphere and non-hard-sphere sources using aqueous silica and polystyrene solutions. Similar aqueous silica solutions. In order to better understand experimental findings and develop more thorough forecasting methods for polydispersed colloidal dispersions, this effort will categorize colloidal suspension viscosity models. Particle buildup is critical in concentrated suspensions because it leads to gelation, as demonstrated by Liang et al. in their study [15]. By changing the antiparticle interactions, concentrated suspensions can have their characteristics and propensity to form aggregates for stabilization changed.

Polymeric microspheres have been used by Applin [16] in a variety of applications, ranging from chromatographic separation methods to the study of air flow through aerodynamic surfaces. Microscopic monodisperse polystyrene was produced utilizing a surfactant-free emulsion polymerization technique (PS). To learn more about the particle size and size distributions, scanning electron microscopy and dynamic light scattering were used to describe the particles. Ifijen [17] states that particle diameter and colloidal stability of composite PS microspheres are influenced by the amount of monomer, amount of initiator, reaction temperature, and stirring rate during polymerization. Spherical particles without aggregation were visible by microscopic examination. The produced PS was fully degraded at 465°C, according to thermogravimetric measurements. With increasing initiator amount, stirring speed, and reaction temperature, Dynamic light scattering (DLS) analysis revealed an average drop in the particle sizes of the synthesized PS microspheres, but the average particle diameter increased with increasing amount of the monomer.

The most effective way to overcome the flocculation resulting from van der Waals attraction is steric stabilization. For example, a brush-like amphiphilic block copolymer can be formed on the particle surfaces either through physical adsorption or chemical grafting [18]. This can be achieved in an aqueous system by changing the temperature and/or pressure to decrease the solvent power of water for stabilizing the moieties. Thermoresponsive ‘switchable’ steric stabilization can be achieved by physical adsorption [19] or chemical grafting of thermoresponsive polymer onto the particle surfaces [20].

This study’s objective was to physically adsorb thermoresponsive PDEGMA polymer onto the surface of PS microspheres in order to synthesize and characterize thermoresponsive colloidal gels. Initially, dispersion polymerization was used to create monodisperse polystyrene microspheres in the size range of 1 to 5 m. Then, in the medium for cell culture, thermoreversible magnertic colloidal suspensions/gels were created by combining various ratios of polystyrene particles (PS) with various quantities of PDEGMA (pre-synthesized using standard free radical polymerization) (DMEM). PDEGMA should be utilized as a steric stabilizer to alleviate the problem of fastness. Moreover, it provides a method of altering the suspension’s properties by serving as a temperature-induced flocculent. As a result, particle gels can be produced by reversibly transforming temperature-stimulated suspensions into particle gels.

## 2. Methods and materials

### 2.1. Materials

Styrene and divinylbenzene (DVB) were washed three times with 5% w/w NaOH to eliminate inhibitors. Then, they were washed with distilled water until the washings were neutral and stored in a refrigerator until use. 2,2-Azobisisobutyronitrile (AIBN) and polyvinylpyrrolidone (PVP K-30, *M*w = 40000) were used as the stabilizer and initiator, respectively. Sigma-Aldrich was used to purchase the macromer di (ethylene glycol) methyl ether methacrylate (DEGMA, Mw = 188) and other compounds. All other solvents, including hexane, ethanol and chloroform, were analytical-grade and utilized exactly as received. It was HPLC grade water.

### 2.2. Synthesis of PS microspheres

Dispersion polymerization was used to create PS microspheres in a sealed round-bottom flask that also had a reflux condenser and a magnetic stirrer. The flask was filled with ethanol (160 g), water (18 g), and PVP (3 g), and the mixture was agitated until the polymer was completely dissolved. The solution was then supplemented with styrene (10 g, 0.096 mol), and DVB (0.5 g, 0.003.9 mol). Nitrogen was pumped into the solution for 30 min, following which the solution was heated to 74°C. Then, AIBN (0.399 g, 0.0024 mol) was added to the solution and the polymerization was continued for 24 h at 120 rpm. The obtained PS microspheres were separated by centrifugation (3000 ×g for 10 min) and repeatedly washed via re-suspension and centrifugation using distilled water. The resulting microspheres were freeze-dried. The percentage yield of the product was calculated by dividing the weight of the freeze-dried polymer by the total weight of the monomers.

### 2.3. Synthesis of linear thermoresponsive ‘control’ polymer

Poly(diethylene glycolmethacrylate) (PDEGMA) was prepared by conventional free radical polymerization in accordance with previous protocols. Diethylene glycolmethacrylate (11 g, 59 mmol) was weighed into a round bottom flask and 1-dodecanethiol (0.08 g, 0.4 mmol) and 20 mL of 2-butanone were added. AIBN (0.07 g, 0.4 mmol) was added and the mixture was degassed with nitrogen for 25 minutes. The solution temperature was elevated to 70°C and polymerization was performed at that temperature for 1 h. The reaction was then stopped by cooling the reaction vessel to 0°C and opening the contents to air. The polymer was precipitated and excess monomers were removed by cycles of precipitation of polymer from 2-butanone into hexane (polymer solution to hexane 1:10). Polymer properties (Mw, Mn), and polydispersity (Ð) were determined by GPC compared to polystyrene standards: Mw = 50.3 kDa, Mn = 28 kDa, Ð = 1.8. NMR data matched those of prior preparations of this material.

### 2.4. Electron microscopy

Particle size distribution, average size, and surface morphology of the fine particles were measured using scanning electron microscopy (SEM) (JEOL 6060 V, JEOL Ltd., Hertfordshire, UK). The samples were prepared on an aluminum stub covered with a carbon tape by permeation of the suspended particles. Smile View (JEOL Ltd., Hertfordshire, UK) was used for particle-size measurements. The average number of diameter (DN), standard deviation (SD), and coefficient of variation (CV) of at least 100 particles from various fields of three photographs of the sample were calculated.

## 2.5. ^1^H NMR spectroscopy

^1^H NMR spectra were recorded in CDCl_3_ on a Bruker 400 spectrometer. The chemical shifts were recorded in ppm relative to the internal standard tetramethyl silane.

### 2.6. Gel permeation chromatography (GPC)

GPC was performed on an instrument (PL-50, Polymer Labs, UK) equipped with a refractive index detector to determine average molecular weight number (*M*n), average molecular weight (*M*w), and polydispersity (Ð) of the synthesized PDEGMA. Two columns (PLgel MIXED-C, 30 cm) were respectively calibrated with PS and chloroform standards. The sample was dissolved in chloroform and was filtered (200 nm filter) before being placed on the column. The process was performed at 40°C with a flow rate of 1 mL/min.

### 2.7. Measurement of cloud point temperatures of thermoresponsive polymers

A thermostat and UV-vis spectroscopy (Beckman Coulter, Model DU 800 UV/VIS Spectrophotometer) were used to measure the cloud point temperature (CPT) of a dilute solution of PDEGMA. The temperature was increased from 10°C to 40°C at 1°C min^−1^ using a Peltier plate. The absorbance of the samples was measured at 550 nm.

### 2.8. Preparation of heat-responsive pendants

The suspensions of varied concentrations (% w/v) of PS microspheres (10%, 15%, 20%, 25%, and 30%) with PDEGMA polymer (1%, 2%, 3%, and 4%) were dissolved in distilled water or cell culture media. Then, they were mixed in a vortex for a minute and cooled to 0°C in an ice bath.

### 2.9. Tube Inversion test

A thermostat bath was used to heat one milliliter of a watery PDEGMA/PS suspension to 37°C for two minutes. This suspension was then put into a 5 mL glass vial. The vial was used to examine the dispersion’s movement, and if the suspended particles did not flow after 10 seconds, the dispersion was assumed to be a gel.

### 2.10. Rheological measurements

An Anton Paar rheometer (MCR301, Anton Paar) was used to perform the rheological measurements. This was equipped with a parallel plate geometry of 25-mm diameter (PP25). The gap between the two plates was fixed at 0.5 mm. The 3.6× software version of Rheoplus was used to analyze the data. The temperature ramp studies that were recorded used a 0.05% strain. From 10 to 40°C, it was heated at a rate of 1 C/min at a frequency of 1 rad/s.

At 10°C, the top and bottom parallel plates were maintained in contact for 5 min before conducting the experiment. Subsequently, a sample of 300 μL was applied to the bottom plate and was equilibrated for 4 min at 10°C. The strain sweep test was conducted on a fresh sample. The samples were placed between the plates at 37°C for 5 min before beginning the strain sweep test. The strain sweeps were conducted at 37°C and an angular rate of 1 rad s^−1^, which yields a strain of 0.01–100%.

## 3. Results and discussion

### 3.1. Synthesis and characterization of PS microspheres

**Figure 1.**
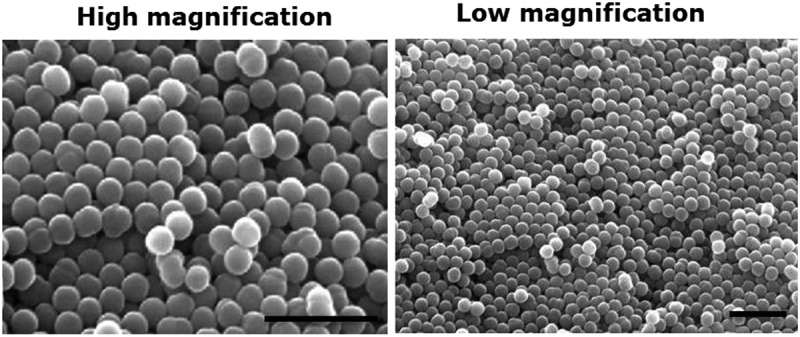
(a) Reaction of styrene and divinylbenzene to produce lightly cross-linked polystyrene (PS) microspheres. (b) Scanning electron micrographs of PS microspheres at high (left) and low (right) magnification; scale bar = 5 µm.

PS microspheres were synthesized by dispersion polymerization method of styrene and DVB in ethanol/water based on a previous report on the synthesis of micron-size monodisperse microspheres [21] (Figure 1a). The particle sizes were 1.5–3.5 μm to avoid potential internalization of the particles when used with mammalian cells [22], while being in the colloidal range to maintain their properties [23].

**Figure:**
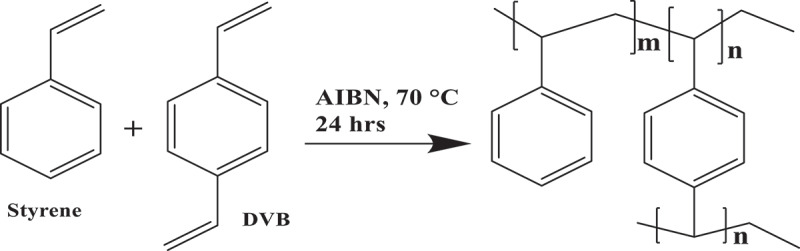

The size distribution and morphology of the PS microspheres were investigated using SEM. The microspheres, shown in (Figure 1b), had an average diameter of 2.5 µm. This size was chosen because microspheres have spherical shapes and can be stabilized without considerable effort (as bigger particles require stabilization). In addition, this size reduces the risk of cell

uptake by smaller particles.

### 3.2. Synthesis and characterization of PDEGMA linear polymer

**Figure 2.**
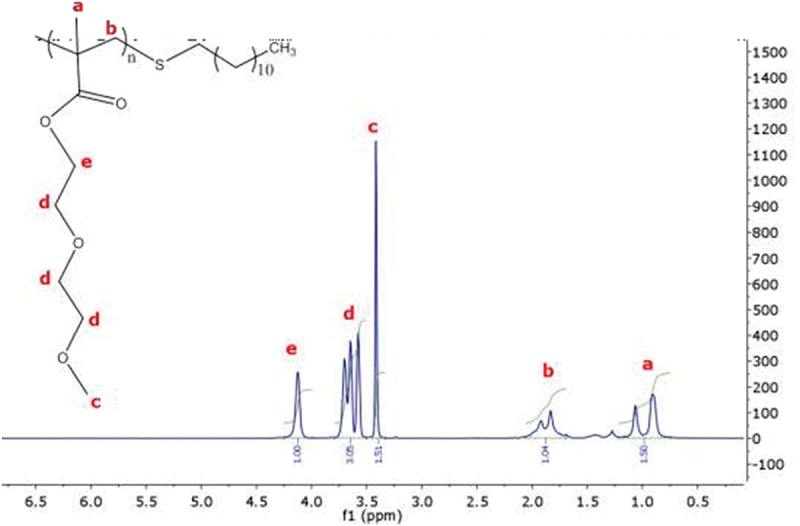
NMR spectrum of the prepared poly(diethyleneglycolmethylmethacrylate) (PDEGMA). and 4% (w/v) PDEGMA.

Thiol, a chain transfer agent that regulates the mass in the free radical polymerization, was used to create PDEGMA thermoresponsive polymers. In order to boost the polymer’s amphiphilicity and hydrophobicity for adsorption onto PS particles, 1-mercaptododecane was used. The synthesized polymer was purified three times by the solvent/anti-solvent technique. The purity of the polymer was measured using ^1^H NMR (Figure 2). The peaks at 5.5–6.5 ppm corresponding to the double bonds of the monomer are absent in this NMR spectra, which confirms the purity of the polymer.

Using GPC, the polymers’ Mn (50838), Mw (28012), and (1.8148) were calculated. The produced polymers were polydisperse, as would be predicted for polymers made using a traditional-free radical approach. The removal of transition metal catalysts, which might be hazardous to cells in biomedical applications, and other potential issues led to the conclusion that controlled-free radical pathways were not required for this polymerization.

**Figure 3.**
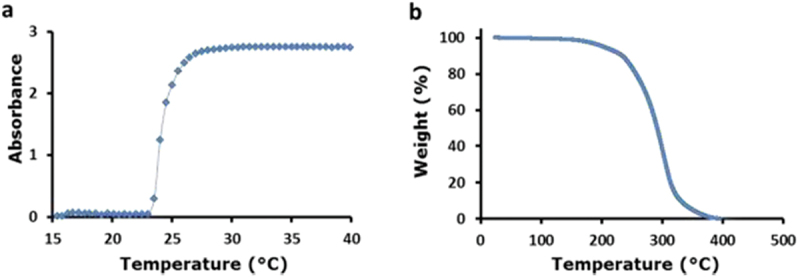
(A) UV-vis spectra of 1% w/v PDEGMA solution in deionized water. LCST behavior was observed at 24°C. All absorbances were measured at a wavelength of 550 nm. (b) Thermogravimetric analysis shows one-step decomposition for PDEGMA polymer, indicating its high purity.

UV-vis spectroscopy was used to confirm the thermoresponsive properties of PDEGMA. CPT was measured by estimating LCST of the linear polymer. The CPT of PDEGMA was measured in deionized water using dilute solutions of PDEGMA (1% w/v), which were heated at 10–40°C. The LCST heat-responsive polymers undergo a coil-to-globule transition and become water insoluble [24]. When polymers break down, their solutions become turbid as a consequence of polymer chain build-up. Turbidity was observed at the CPT. The PDEGMA solution showed a marked increase in absorbance at 24°C (Figure 3), which indicates the CPT of the free polymer resulting from pronounced chain collapse and aggregation of polymer chains.

The synthesized polymer was further characterized using thermogravimetric analysis to confirm the structural components and purity. A one-step weight loss of 100% was observed between 200°C and 380°C corresponding to the decomposition of the PDEGMA linear polymer (Figure 3). This indicates the high purity and lack of contamination in the polymers.

### 3.3. Colloidal gel evaluation

Thermoresponsive suspensions were obtained by mixing different w/v percentages of PS microspheres with the solution of PDEGMA dissolved in complete DMEM medium or in deionized water. Strong adsorption of polymer chains onto the particle surfaces is required for effective steric stabilization. This induces effective repulsions and high surface coverage, resulting in good solvency of the stabilizing chains. In addition, a low concentration of free polymer should be ensured to avoid depletion attraction through bombarding microparticles by the surrounding collapsed chain-free polymers in the solution above the LCST [24]. Thus, when the thermoresponsive polymer was adsorbed on microspheres below its LCST, it can act as a steric stabilizer and avoids interparticle association. However, above the LCST, particle destabilization and flocculation can occur as the adsorbed polymer chains collapse. Particle interaction is important for aggregation of particles and gelation. Gelation occurs only if sufficient number of particles have strong interparticle attractions. For instance, if space-filling networks of particle flocs are in sufficient strength and number, a space-filling particle gel is formed.

**Figure 4.**
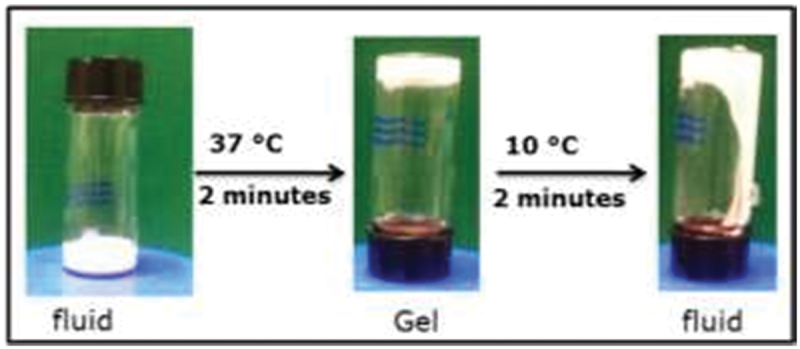
Images of tube inversion test of the suspension containing 25% (w/v) PS particles and 4% (w/v) PDEGMA.

PDEGMA behaves as an amphiphile below its LCST owing to its hydrophilic ethylene glycol side chains and hydrophobic methacrylate backbone. Consequently, the methacrylate backbone and the dodecane thioether chain terminus is adsorbed onto the surface of the PS microparticles in the suspension. As the solvent quality of PDEGMA as a steric stabilizer is temperature dependent, a temperature greater than its LCST will alter the bonding behavior of the coated particles in the suspension. Water is a good solvent for PDEGMA below its LCST. At this temperature, the contact between PDEGMA with the solvent (water) molecules is enthalpically favored, which causes the polymer chains to swell and move away from each other. However, these polymeric segments break down or undergo a coil-to-globule transition above the LCST when water becomes a poor solvent. Large 3D particle clusters appear as a result of mutual intermolecular attractions, causing build-up of polymeric associations between the particles. We observed that the sterically stabilized PS particles bearing PDEGMA in a suspension did not interact under the LCST. However, over the LCST, the degree of shear thinning increased because of strong interparticle attractions, resulting in structural buildup of the particle networks and viscosity. Furthermore, over the LCST, PDEGMA reduces into a globular conformation and its hydrophobicity controls the interactions between particles, which converts the distorted layers into a gel. Figure 4 shows the changes in the colloidal fluid-gel with changing temperature in plain-tube inversion tests. Several tube inversion assay experiments were performed using different weight combinations (concentrations) of PDEGMA and PS particles. Space-filling gels are typically formed at 37°C. In order to prepare PDEGMA solutions, a cell culture medium and deionized water were combined. The concentration of PDEGMA in the suspensions ranged from 1% to 4% (w/v) in order to control the minimum (critical) concentration of PS particles required to form gels at 37°C. Space-filling gels become heavy without synergy in the tube inversion. Gels were only formed at a PS concentration higher than 10% (w/v) combined with 4% (w/v) PDEGMA at 37°C. At PS concentration less than 10% (w/v), only particle accumulation was observed. If the PDEGMA content is reduced to <3% (w/v), the required PS concentration for gelation increased to 15% (w/v). These results confirm that the suspensions become gels when the minimum particle concentration is 10% PS with 3% or 4% PDEGMA. However, suspensions containing 1% or 2% PDEGMA required 15% PS particle fraction for gelation. In addition, the maximum concentration of particles that could be loaded was 25%, as concentrations greater than this number resulted in the formation of a paste and did not flow at any temperature. The tube inversion results showed no obvious difference between the thermoreversible gelation in water and culture medium. Hence, all experiments were conducted in culture medium. The gelation is particle dependent because no thermoreversible gelation was observed for the suspensions that were made from only PDEGMA without the PS particles.

### 3.4. Rheological studies

Particle gels are viscoelastic materials as they exhibit viscosity and an elastic response to an applied force. Therefore, rheological measurements were performed to investigate the viscoelastic and mechanical properties of the thermoresponsive colloidal suspensions/gels. PS microspheres (2.5 µm) were present in all suspension/gels. Therefore, to investigate the temperature-dependent viscoelastic characteristics of aqueous PS, temperature ramp tests were performed. Tests were performed for suspensions which contained varying concentrations of thermoresponsive polymer and/or no polymer, while maintaining the concentration of the PS particles at 25% (w/v).

**Figure 5.**
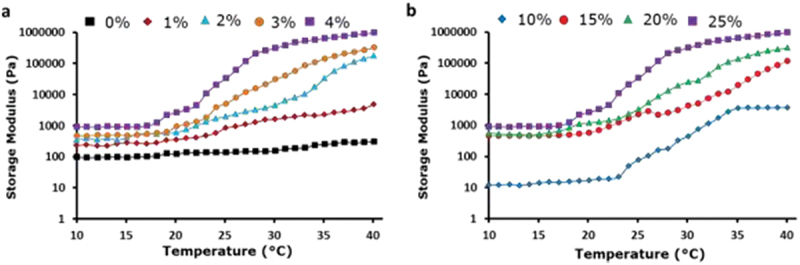
Temperature sweep experiments reveal differences in temperature and G′ of PS/PDEGMA or PS particulate suspensions at (a) different PDEGMA concentrations and constant PS particle concentrations of 25%; and (b) varying particle concentrations and a PDEGMA concentration of 4%.

As shown in Figure 5, all suspensions containing PDEGMA polymer exhibited the temperature dependence of shear modulus (G′) above 20°C and this increase in G′ values is proportional to the amount of PDEGMA in the suspension. In contrast, PS particle suspensions lacking PDEGMA exhibited no temperature-dependent modulus of elasticity for indoor temperatures. At temperatures below 22°C, both G′ values remained low and nearly constant, indicating that the suspensions remained in the fluid (aggregated) state. At temperatures above 22°C, G′ values increased non-linearly with increasing temperature. However, G′ values increased with temperature, consequently increasing interparticle attraction. Similarly, with increasing temperature, dehydration of the adsorbed PDEGMA layers increased, causing stronger hydrophobic interactions and antiparticle associations. Therefore, increasing the concentration of either thermoresponsive polymers and/or particles alters the thermogelation behavior and mechanical properties of the analyzed suspensions. In addition, gelation required higher temperatures and lower particle concentration. High temperature causes strong interparticle attractions and a strong particle network, resulting in more elastic gels. In contrast, at temperatures over the LCST, the interparticle attraction is weak, resulting in extra particles creating dense systems of satisfactory elastic strength [25].

A strain sweep test was conducted to observe the mechanical properties and linear viscoelastic region control (LVR) of the prepared gel. G was stress-free in this region and breaks down into a gelatinous structure outside this region, which was evident from the immediate decline in G at higher distortions. The jelly was converted to a near-liquid substance at its critical strain point (tan δ = 1). The LVR depended on the concentration of the small particles. The distortion in the strain sweep tests is plotted against elastic (G′) modulus. The critical strain, which is frequently taken as the strain at which tan δ equals 1 or when G′ and G′′ crossover occurs.

**Figure 6.**
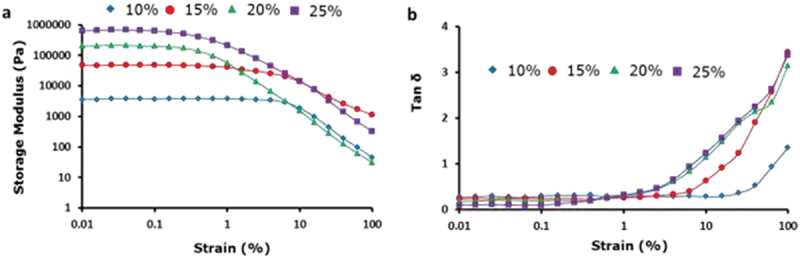
Stress sweep tests display the dissimilarity of (a) G′ and (b) strain at PS particle concentrations at a rate of 1 rad s^−1^ and 37°C with tan δ.

Figure 6 shows the strain sweep tests of PS/PDEGMA particulate suspensions gelled at 37°C. A small LVE value is shown for all types of gels that have a critical strain amount of ~1%. Because the particles are fewer, they were susceptible to deformation than single-chain polymer molecules. Moreover, the strands of aggregated particles are less elastic. The macromolecular gels tended to be less brittle in comparison to particulate gels, and the amount of the critical strain is below 1%.

G′ was free from strain (<0.5%) at all concentrations (Figure 6). This suggests that the gels were in a linear viscoelastic system. The LVE region shortened with increasing particle concentration, which is attributed to an increase in the solid particle content that makes the jelly delicate and less viscous. MPS particles were more elastic but slightly more brittle. These networks of flocculated particles are not flexible and, hence, affected the overall flexibility of the gels [25].

## 4. Conclusion

Thermoresponsive colloidal gels were synthesized via physical adsorption of PDEGMA onto the surfaces of PS microspheres. Our results demonstrated the potential of these colloidal gels for use as a 3D scaffold in cell expansion. Moreover, rheometric analysis demonstrated that the prepared colloidal gels have mechanical and viscoelastic properties suitable for use as scaffolds for cell-culture applications. Furthermore, this strategy is not complex or time-consuming and can be adjusted to various microparticle surfaces.

## Data Availability

The data that support the findings of this study are openly available in [repository name] at [URL], reference number [reference number].
